# Role of glial cells in motor neuron degeneration in hereditary spastic paraplegias

**DOI:** 10.3389/fncel.2025.1553658

**Published:** 2025-04-15

**Authors:** Manaswini Vijayaraghavan, Sarvika Periyapalayam Murali, Gitika Thakur, Xue-Jun Li

**Affiliations:** ^1^Department of Biomedical Sciences, University of Illinois College of Medicine Rockford, Rockford, IL, United States; ^2^Department of Bioengineering, University of Illinois at Chicago, Chicago, IL, United States

**Keywords:** axonal degeneration, motor neurons, astrocytes, microglia, lipid dysfunction, neuroinflammation

## Abstract

This review provides a comprehensive overview of hereditary spastic paraplegias (HSPs) and summarizes the recent progress on the role of glial cells in the pathogenesis of HSPs. HSPs are a heterogeneous group of neurogenetic diseases characterized by axonal degeneration of cortical motor neurons, leading to muscle weakness and atrophy. Though the contribution of glial cells, especially astrocytes, to the progression of other motor neuron diseases like amyotrophic lateral sclerosis (ALS) is well documented, the role of glial cells and the interaction between neurons and astrocytes in HSP remained unknown until recently. Using human pluripotent stem cell-based models of HSPs, a study reported impaired lipid metabolisms and reduced size of lipid droplets in HSP astrocytes. Moreover, targeting lipid dysfunction in astrocytes rescues axonal degeneration of HSP cortical neurons, demonstrating a non-cell-autonomous mechanism in axonal deficits of HSP neurons. In addition to astrocytes, recent studies revealed dysfunctions in HSP patient pluripotent stem cell-derived microglial cells. Increased microgliosis and pro-inflammation factors were also observed in HSP patients’ samples, pointing to an exciting role of innate immunity and microglia in HSP. Building upon these recent studies, further investigation of the detailed molecular mechanism and the interplay between glial cell dysfunction and neuronal degeneration in HSP by combining human stem cell models, animal models, and patient samples will open avenues for identifying new therapeutic targets and strategies for HSP.

## 1 Introduction to hereditary spastic paraplegia

Hereditary spastic paraplegias (HSPs) are a group of neurodegenerative diseases characterized by axonal degeneration affecting the corticospinal pathways, leading to lower limb stiffness and weakness (Blackstone et al., 2011; Fink, 2013). There are over 80 identified genomic loci or genes associated with HSP. Based on the inheritance pattern, HSPs are classified as autosomal dominant (AD), autosomal recessive (AR), X-linked recessive, and mitochondrial inheritance (Finsterer et al., 2012). HSPs are also categorized as pure or complicated depending on their clinical manifestation. Pure forms of HSP patients exhibit clinical signs predominantly associated with deficits of the corticospinal tract, including lower limb weakness and stiffness, disruption in vibration perception and proprioception, and fluctuating hypertonic urine disturbance. In addition to corticospinal tract deficits, complex forms of HSP patients accompany additional neurological symptoms including ataxia, thin corpus callosum, extrapyramidal symptoms, chorioretinal degeneration, peripheral neuropathy, and mental impairment (Finsterer et al., 2012; Murala et al., 2021).

### 1.1 Epidemiology of HSP

For both AD and AR forms, the estimated prevalence of HSP globally is 1.8/100,000 patients. The prevalence rate varies according to the patient’s inclusion criteria, diagnosis, categorization, and geographic area (Chrestian et al., 2017; Koh et al., 2018). Three to ten HSP cases are thought to exist for every 100,000 people in Europe. It is believed that for every 100,000 people in Japan, there are 0.2 instances. The prevalence of AR-HSP varies from 0.0 to 5.3/100,000 population, with a pooled average of 1.8/100,000, while that of AD-HSP is from 0.5 to 5.5/100,000 (Ruano et al., 2014; de Souza et al., 2017). Common AD forms of HSP include SPG3A, SPG4, and SPG31, which are caused by mutations in *ATL1, SPAST and REEP1* (Finsterer et al., 2012; Hedera, 1993). SPG7, SPG11, and SPG15 are common autosomal recessive forms (Finsterer et al., 2012; Stevanin et al., 2008), and patients commonly exhibited additional clinical symptoms (complex form) in addition to muscle weakness and spasticity (Table 1).

**Table 1:** Overview of common HSP types, genes, protein involved and inheritance pattern.

Gene	HSP designation	Protein involved	Type of HSP	Inheritance pattern	Function
*PLP1*	SPG2 (Saugier-Veber et al., 1994; Yao et al., 2023)	Proteolipid protein 1	Pure and complex	X-linked recessive	Major component of myelin
*ATL1*	SPG3A (Zhao et al., 2001; Khan et al., 2014)	Atlastin-1	Pure	Autosomal dominant	Endoplasmic reticulum (ER) morphogenesis and axon elongation
*SPAST*	SPG4 (Hazan et al., 1999; Solowska and Baas, 2015)	Spastin	Pure	Autosomal dominant	Microtubule-severing protein
*SPG7*	SPG7 (Casari et al., 1998; Pfeffer et al., 2015)	Paraplegin	Pure and complex	Autosomal recessive	Key component of the “m-AAA protease” complex, misfolded protein degradation and ribosome assembly
*SPG11*	SPG11 (Stevanin et al., 2007; Goizet et al., 2009)	Spatacsin	Complex	Autosomal recessive	Autophagy lysosomal reformation, mitochondrial dynamics
*ZFYVE26*	SPG15 (Hanein et al., 2008; Marrone et al., 2022)	Spastizin	Complex	Autosomal recessive	Autophagy lysosome reformation
*SPG20*	SPG20 (Patel et al., 2002; Dardour et al., 2017)	Spartin	Complex	Autosomal recessive	Lipid droplet turnover
*ACP33*	SPG21 (Simpson et al., 2003; Soderblom et al., 2010)	Maspardin	Complex	Autosomal recessive	Intracellular trafficking and protein interactions in neurons
*REEP1*	SPG31 (Zuchner et al., 2006; Lim et al., 2015)	Receptor expression – enhancing protein 1	Pure	Autosomal dominant	Endoplasmic reticulum network
*FA2H*	SPG35 (Edvardson et al., 2008; Rattay et al., 2019)	Fatty acid 2 – hydroxylase	Complex	Autosomal recessive	Enzyme essential for producing 2-hydroxy glycosphingolipids, key myelin components
*PNPLA6*	SPG39 (Fink, 2013; Read et al., 2009)	Neuropathy target esterase	Complex	Autosomal recessive	ER-localized phospholipase B that regulates lipid levels
*C19orf12*	SPG43 (Meilleur et al., 2010; Landoure et al., 2013)	Mitochondrial membrane protein associated with neurodegeneration	Complex	Autosomal recessive	Lipid metabolism and mitochondrial function
*AP4B1*	SPG47 (Blumkin et al., 2011; Wiseman et al., 2024)	AP-4 complex subunit beta-1	Complex	Autosomal recessive	AP4B1 is a subunit of AP-4, forming a protein complex that regulates the transport of membrane proteins
*AP4M1*	SPG50 (Bellad et al., 2021)	AP-4 complex subunit mu-1	Complex	Autosomal recessive	AP4M1 is an integral subunit of the AP4
*AP4S1*	SPG52 (Tessa et al., 2016)	AP-4 complex subunit sigma-1	Complex	Autosomal recessive	Protein trafficking from the trans-Golgi to the endosomal-lysosomal system
*CYP2U1*	SPG56 (Yang et al., 2016; Minase et al., 2017)	Cytochrome P450 2U1	Complex	Autosomal recessive	Lipid metabolism, fatty acid hydroxylation

### 1.2 Neuropathology of HSP

HSPs are caused by the degeneration of cortical motor neuron axons, which interrupts the signaling transmissions to lower motor neurons (located in brainstem and spinal cord) and subsequent muscle cells, leading to the atrophy and weakness of muscles (Blackstone, 2018; Soderblom and Blackstone, 2006). Axon degeneration involving the lateral corticospinal pathways was most frequently observed in postmortem investigations of HSP patients. The degeneration was most severe in the lumbar and thoracic region of the spinal cord and comparatively mild in the cervical section. Fasciculus gracilis fibers are known to exhibit axonal degradation, which is particularly prominent in the cervical region of the spinal cord. The degree of demyelination in these fibers is directly proportional to the degree of axon degeneration, rather than indicating a fundamental demyelinating reason for the condition (Fink, 2013). HSP axonopathy exhibited a particular vulnerability to long motor and sensory axons. Peripheral neuropathy is a prevalent symptom of several subtypes of HSP, which extends beyond the central nervous system (Fink, 2013).

Axons are myelinated by oligodendrocytes in the central nervous system. Because of the degeneration of corticospinal tracts, myelin abnormalities are commonly observed in patients with HSP (Fink, 2013; Blackstone, 2012). In some HSP patients, myelin abnormalities could be primary and precede to axonal degeneration. For example, mutations in proteolipid protein 1 (*PLP1*), a major component of myelin, underlie type 2 spastic paraplegias (SPG2) (Khalaf et al., 2022) (Table 1). Moderate cerebral atrophy, extensive pallor of CNS myelin (corticospinal tract), and significant axonal degeneration of the corticospinal tract were seen postmortem in an SPG2 HSP (Suzuki et al., 2011). PLP1 is highly expressed in oligodendrocytes, implying the contribution of glial cells in the pathogenesis of HSP. In addition to oligodendrocyte, recent studies revealed the critical role of other types of glial cells especially astrocytes (Mou et al., 2020) in the degeneration of cortical motor neuron axons in HSP, providing new targets for developing therapies for HSP.

## 2 Astrocytes: a novel player in the pathogenesis of neurodegeneration and HSP

Astrocytes, a major type of glial cell, constitute about 20–40% of the total cells in the mammalian brain (Khakh and Sofroniew, 2015; Liddelow and Barres, 2017). Astrocytes have long been considered as the major supporting cell for neurons, though more and more studies show their critical roles in neurodevelopment and diseases. They play a pivotal role in maintaining cell homeostasis and blood brain barrier (BBB) integrity, secreting trophic molecules, and regulating the brain microenvironment. Apart from all these, astrocytes also aid in a normal synaptic function and transmission (Farhy-Tselnicker and Allen, 2018). Astrocytes, the most ubiquitous cell type, can form bidirectional contact between the neurons as well as the microvascular endothelial cells lining the cerebral blood vessels. Through this interaction, astrocytes carry glucose, oxygen, and other substances from blood to the neurons thus providing nutritional support for the neurons (Sofroniew and Vinters, 2010). In reaction to neuronal activity, astrocytes can alter local blood flow by producing vasodilators like nitric oxide or arachidonic acid. This increases blood flow and, thus, provides active neurons with nutritional support (Lovick et al., 2005). In addition to the regulation of blood flow, astrocytes also transfer mitochondria to neurons (Khakh and Sofroniew, 2015; Kim et al., 2019). Furthermore, four separate subtypes of anatomically and structurally diverse GFAP+ astrocytes—interlaminar, protoplasmic, varicose, and fibrous astroglia—can be found in the human brain (Oberheim et al., 2012; Vasile et al., 2017). Interlaminar astrocytes are found in layer 1 of the cerebral cortex and extend long, varicosity-free processes into deeper layers. The most prevalent astrocytes in gray matter, protoplasmic astrocytes, are found in layers 2–6 of the cortex, while varicose astrocytes are located in the white matter and layers 5 and 6 (Degl'Innocenti and Dell'Anno, 2023). Fibrous astrocytes align with axon fibers and terminate at Ranvier’s nodes. The detailed functions of these astrocytes and whether they play differential roles in pathological changes remains to be investigated.

As a reaction to the CNS damage, astrocytes undergo changes to become reactive astrocytes (Liddelow and Barres, 2017; Ding et al., 2021). Reactive astrocytosis is primarily characterized by cellular hypertrophy, altered gene expression and possibly astrocyte proliferation. Reactive astrocytes alter their molecular expression, and a wealth of information is known about the chemicals that can cause reactive astrogliosis. Through reactive astrogliosis, astrocytes react to brain injury or neurodegeneration by producing extracellular molecules such cytotoxins (e.g., LCN2), inflammatory factors (e.g., IL-1β, TNF-α, nitric oxide), and neurotrophic factors (e.g., BDNF, NGF). These molecules influence the CNS in a neuroprotective or neurotoxic way (Sofroniew and Vinters, 2010). Reactive astrocytes can be classified as either neurotoxic (A1) or neuroprotective (A2) based on their gene expression patterns and secreted factors (Lawrence et al., 2023; Fan and Huo, 2021). In reaction to acute damage such as ischemia and tissue injury, astrocytes can adopt a neuroprotective A2 phenotype and produce neurotrophic factors, such as BDNF and NGF, to promote recovery (Liddelow and Barres, 2017; Qian et al., 2018). Conversely, chronic damage and neurodegeneration are associated with a cytotoxic A1 reactive astrocyte phenotype. Pro-inflammatory factors generated from blood–brain barrier damage and microglial activation, which will be discussed in the following sections, are common stimuli for A1 toxic astrocytes (Liddelow et al., 2017; Geng et al., 2025). At the same time, inflammatory indicators such as nitric oxide, IL-1β (interleukin 1 beta), and TNF-α (tumor necrosis factor alpha) are further upregulated by A1 astrocytes (Lawrence et al., 2023; Liddelow et al., 2017), exacerbating neuroinflammation and resulting in aberrant synaptogenesis and neuronal degeneration.

Astrocytes are a major component of blood brain barrier (BBB). The BBB, also known as the neurovascular unit, is made up of a layer of astrocytes, pericytes, and microvascular endothelial cells that surround cerebral capillaries. It keeps the susceptible central nervous system safe from external threats while maintaining an appropriate blood supply. Tight junctions between endothelial cells preserve the BBB’s low permeability, which is used to tightly control what enters the brain parenchyma. Its ability to operate properly depends on the close interactions between thick astrocyte processes, or end feet, which encase blood vessel endothelial cells and create the glial limiting membrane, a charged basement membrane (Tabata, 2015; Liu et al., 2020). The integrity of the outer and inner cerebrospinal fluid-brain barriers, which divide the brain parenchyma from the ventricles and subarachnoid space, respectively, is also preserved by astrocytes. The BBB plays a vital function in preventing peripheral immune cells from extensively entering the central nervous system. When immune cells infiltrate due to compromised barrier integrity, they produce pro-inflammatory cytokines and harmful reactive oxygen species (ROS), which can cause neuroinflammation and impair neuronal function, further exacerbating neurodegeneration (Lawrence et al., 2023; Whish et al., 2015; Brochner et al., 2015). Though it has been shown that astrocytes have a critical role in disease progression and motor neuron degeneration in ALS cell and animal models (Van Harten et al., 2021; Pehar et al., 2017), the effects of astrocytes on neurons and their contribution to the pathogenesis of HSP had remained unknown. Using human stem cell-based models of SPG3A, a recent study reported the lipid deficiency caused by *ATL1* mutations lead to axonal degeneration of human cortical projection neurons (Mou et al., 2020), providing evidence to support the direct interactions between neuron and astrocytes in HSP human models, opening new opportunities for studying HSP. This mini-review highlights recent advances in HSP, while additional information on other motor neuron diseases has been summarized in previous works (Ravi et al., 2021; Yamanaka and Komine, 2018; You et al., 2023; Fernandez-Lizarbe et al., 2019; Provenzano et al., 2023; Olympiou et al., 2016).

## 3 Role of astrocytes in neuronal degeneration associated with HSP: impaired lipid metabolism in SPG3A astrocytes underlies the degeneration of cortical projection neurons

SPG3A is the most common dominant form of HSP, caused by the mutations in the *ATL1* gene which encodes Atlastin-1 protein. Atlastin 1 is a membrane bound ER-localized dynamin like GTPase (Zhao et al., 2001). *ATL1* mutations impair the formation of a three way ER tubule junctions which leads to improper tubule and vesicle formation (Orso et al., 2009; Zhao et al., 2016). Apart from SPG3A protein, Atlastin 1, several other HSP related proteins like spastin (SPG4), seipin (SPG17), spartin (SPG20), and REEP1 (SPG31) were also found to be participating in regulating the number and size of the lipid droplets in HEK293, COS7 and HeLa cells *in vitro* (Klemm et al., 2013; Eastman et al., 2009; Fei et al., 2011; Papadopoulos et al., 2015; Renvoise et al., 2012). These forms of HSP cover over 50% of HSP patients, suggesting lipid dysfunction as a common pathological change for HSP patients.

Using astrocytes derived from SPG3A patient iPSCs, Mou et al. reported reduced size of lipid droplets in SPG3A astrocytes (Mou et al., 2020). Moreover, this study further showed that the efflux of cholesterol from SPG3A astrocytes is significantly reduced. Moreover, restoring the cholesterol transfer using LXR agonists mitigates axonal degeneration of cortical projection neurons. Thus, these data demonstrate a novel role of astrocytes in HSP and provide evidence of the impaired cholesterol trafficking between SPG3A patient iPSC-derived astrocytes and neurons.

Cholesterol is crucial for normal axonal functions and is highly concentrated in the brain (Mauch et al., 2001; Pfrieger, 2003), primarily due to synthesis in glial cells since the blood–brain barrier blocks lipoprotein cholesterol uptake. After birth, neurons depend on glial-supplied cholesterol. Issues with lipid transfer from glial cells to neurons might contribute to neurological diseases (Dietschy, 2009; Zhang and Liu, 2015). In SPG3A stem cell-derived astrocytes, mutations in *ATL1* disrupt lipid metabolism as indicated by lower levels of PLIN2, NR1H2, and SREBP1 (Mou et al., 2020; Shimano and Sato, 2017), leading to smaller lipid droplets (LDs) in astrocytes and reduced cholesterol transfer. Treatment with GW3965 mitigates these deficits and rescues subsequent axonal degeneration of SPG3A cortical neurons. The proposed model suggests that *ATL1* mutations impair lipid biogenesis and cholesterol trafficking in glial cells, causing lipid dysfunction and axonal degeneration in cortical motor neurons. Moreover, reduced cholesterol and axonal defects can be corrected by adding cholesterol or using conditioned media from healthy astrocytes (Mou et al., 2020), revealing a non-cell autonomous mechanism where glial dysfunction affects neuronal health.

**Figure 1:**
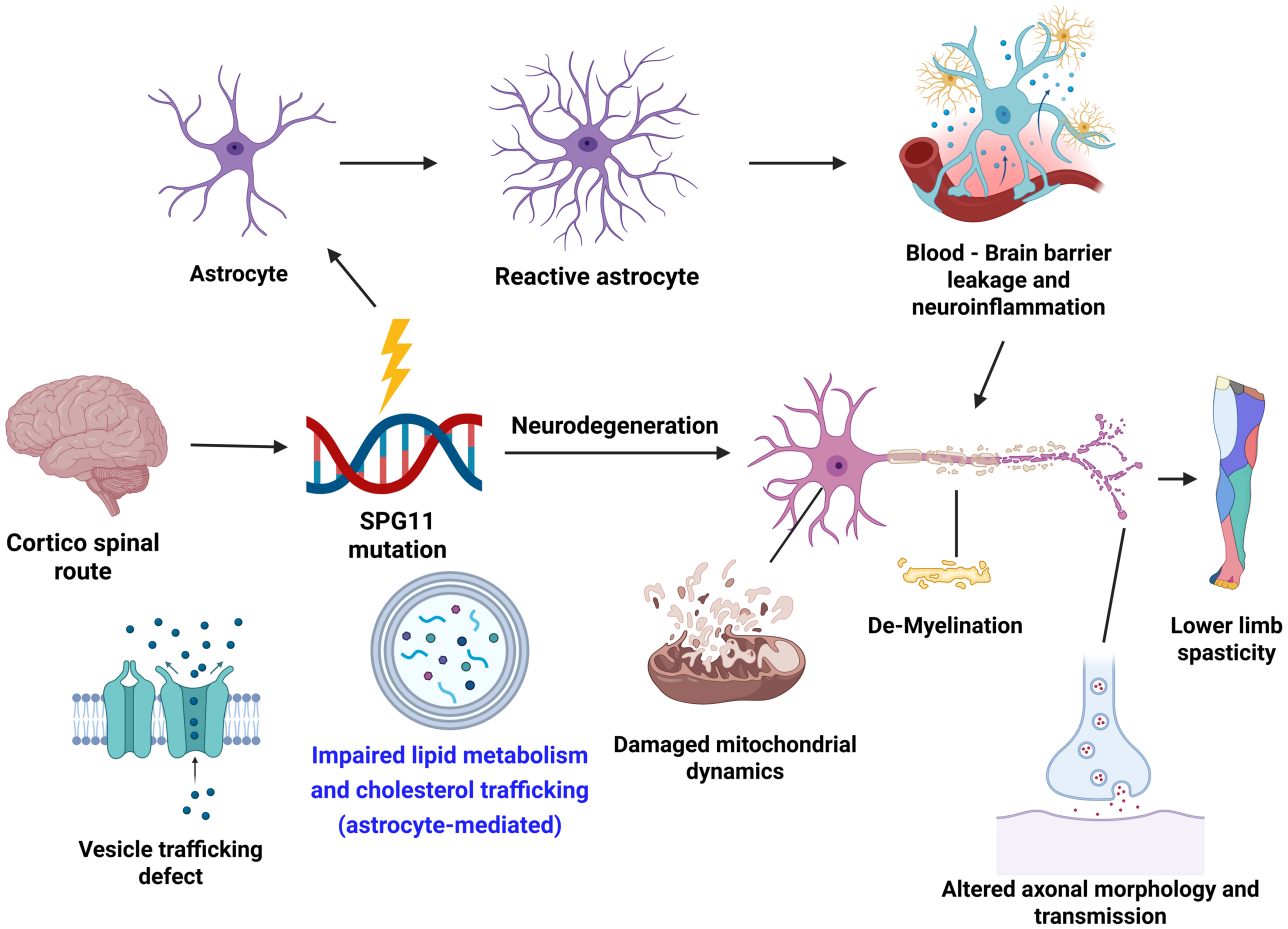
Astrocytes and major pathways affected in the disease mechanism of hereditary spastic paraplegia (HSP). Created in BioRender. Li, X. (2025) https://BioRender.com/7uxkfnq.

In addition to SPG3A, lipid and cholesterol trafficking deficits have been reported in other forms of HSP (Marrone et al., 2022; Gao et al., 2024). Interestingly, the cholesterol trafficking deficits can also be seen in neurons as indicated by the accumulated cholesterol in the lysosomal of neurons (Chai et al., 2023). Whether cholesterol trafficking is also impaired in astrocytes in SPG11, and how the interaction between neuron and astrocytes deficits in the axonal degeneration in different forms of HSP are interesting questions and await further investigation. Lipid dysfunction can also affect other pathological processes, including vesicle trafficking and mitochondrial dynamics (Figure 1). Moreover, reactive astrocytes in response to degeneration could lead to BBB leakage and neuroinflammation. Further research to investigate the interplay between these processes in the pathogenesis of HSP, as well as similarities and differences between HSP and other motor neuron diseases, will provide valuable insights and new therapeutic strategies for rescuing disease phenotypes and developing therapies for HSP.

## 4 Impact of microglial cells on HSP

Microglia, also known as resident brain macrophages, are glial cells that are crucial to the CNS’s immunological processes. Microglia are derived from the mesoderm, and their precursor cells in the yolk sac are called erythromyeloid progenitors (EMPs). This contrasts with neurons, astrocytes, and oligodendrocytes, which come from the ectoderm (Nakaso, 2024). Microglia plays a crucial role in phagocytosing foreign materials and other dead substances and induces immune responses in the brain (Prinz et al., 2021). Apart from phagocytic activity, activation of microglia leads to the release of inflammatory mediators including both pro-inflammatory cytokines and chemokines, further exacerbating the degeneration of neuronal cells (Rodriguez-Gomez et al., 2020). Microglia are observed to be in a ramified structure with elongated protrusions in their resting state. During nerve injuries or any other neurological diseases, the microglia are activated wherein, the microglia undergo morphological changes exhibiting an enlargement in the cell body and shrinking of the protrusions.

The mechanism of microglial activation can be diverse and complex based on the progression of the neurological disease and the region of the brain affected (Nakaso, 2024). The hallmarks of neurodegenerative diseases include a gradual, long-term loss of neurons and abnormally elevated levels of cytotoxic components, such as extracellular debris and reactive oxygen species. These damage-associated molecular patterns (DAMPs) can activate and recruit neurotoxic microglia through binding and activating the Toll-like receptors on microglia. DAMPs can also activate the nuclear factor kappa B (NF-κB) pathway, leading to the activation of microglia cells and the release of pro-inflammatory cytokines (e.g., IL-1β, IL-6, TNF-α) (Biswas, 2023). One important biochemical pathway during microglial activation is tyrosine phosphorylation, which has been shown to be enriched in microglial cells after neurotoxicity (Lull and Block, 2010). Protein kinase C (PKC) also contributes to microglial cytokine production by boosting TNF-α release (Gordon et al., 2016). More research is required to identify the detailed cellular and biochemical mechanisms underlying microglia activation to better understand its role in neurodegenerative diseases.

A recent study using a Spg11^−/−^ mouse model found that neuroinflammatory changes, indicated by CD8+ T lymphocytes invading the brain, exacerbated neurodegeneration (Horner et al., 2022). Exactly how CD8+ T-cell invasion takes place in Spg11^−/−^ mice remains uncertain. Microglia have been demonstrated to be activated in several brain regions, which might potentially boost the attraction of pathogenic CD8+ T cells. By combining iPSC-derived microglial cells, patient post-mortem brain sections, and immune-analyses of patient peripheral blood cells, Krumm et al. revealed the novel role of innate immunity in neuroinflammation and neurodegeneration in SPG11-HSP (Krumm et al., 2024). Extensive and severe microgliosis was seen in the postmortem brain tissues from SPG11–HSP, and the disease-associated microglia marker was expressed. Interestingly, higher blood serum levels of proinflammatory monocytes and the proinflammatory cytokine IL-6 were observed in SPG11 patients. Moreover, the activation of interferon gamma (IFNγ) was revealed in patient induced pluripotent stem cell-derived microglia-like cells, as well as in SPG11^−/−^ mouse model and the SPG11 postmortem tissues (Krumm et al., 2024). Further analysis of patient stem cell-derived microglial cells reveals that the IFNγ-STAT1 mediated pathway underlies the neuroinflammation and the degeneration of neurons in SPG11. Interestingly, direct target T-cell-based response using immunomodulator also showed protective effects in SPG11 animal models (Horner et al., 2022; Horner et al., 2024), suggesting that T-cell-mediated immune response contributes partially to neuroinflammation. The detailed mechanisms and interplay between the microglia- and T cell-mediated immune responses, as well as the role of neuroinflammation in SPG11 and other forms of HSP are valuable questions to be addressed to fully understand the therapeutic potential in targeting glial cells and neuroinflammation in HSP.

## 5 Discussion

A challenge in studying glial cells in HSP using human pluripotent stem cell models is achieving effective differentiation and maturation of glial cells. Establishing consistent and physiologically relevant cell populations through reproducible protocols is vital. By combining critical morphogens and chemicals to recapitulate developmental cues, the paradigms to efficiently differentiate astrocytes and microglial cells have been established (Tao and Zhang, 2016; Teo et al., 2024; Douvaras et al., 2017; Krencik et al., 2011). Further efforts are needed to generate sub-populations and region-specific astrocytes and microglia from human pluripotent stem cells. Another important area is to promote the maturation and function of glial cells, and one way to achieve this is to co-culture glial cells with neurons and/or other types of cells.

The development of co-culture systems with neurons is essential to replicate the cellular complexity and interactions that affect disease progression. Co-culture and triple culture systems serve as *in vitro* models that simulate the interactions among various cell types (neurons, astrocytes, microglia, and oligodendrocytes). These systems provide enhanced physiological relevance and help elucidate numerous biological processes such as differentiation, proliferation, migration, and signaling pathways.

To better mimic the *in vivo* microenvironment, extracellular matrix, growth factors and other cellular components could be used to promote the maturation and function of the co-cultures. Another strategy is to build 3-dimensional (3D) organoids to model the complex brain environment. Given that microglial cells are derived from different germ layers and are usually absent in traditional cortical organoids, generating organoids enriched in microglial cells (or endothelial cells, major components for neurovascular structures) and then assembling them with cortical organoids (which contain neurons and glial cells) could overcome this challenge. Organoids serve as powerful 3D models derived from stem cells, offering a more physiologically relevant system mimicking human tissue complexity. The 3D organoid culture also promotes the maturation of glial cells, allowing the study of these cells in the development and progression of diseases (Amin and Pasca, 2018; Lanjewar and Sloan, 2021). Using 3D cultures of cells derived from patients with Charcot–Marie–Tooth disease (CMT2A), a recent study generated organoids containing myelinating Schwann cells, enabling the testing of PMP22 inhibitors and the study of cell interactions in peripheral neuropathies (Van Lent et al., 2023). The organoid models offer unique human-relevant systems for exploring cellular interactions and testing therapeutics for neurodegenerative diseases, including HSP. The 3D printing of different types of cells provides another valuable approach to study the complex interactions between different cell types in the pathogenesis of HSP.

Further research into the role of glial cells in the pathogenesis of HSPs will test not only individual glial cell types, but also the interplays between glial cells and neurons, and between different pathological processes. Effective therapeutic approaches should address both neuronal and glial dysfunctions, and the combination of glial-targeted therapies with neuronal interventions may offer synergistic benefits. Furthermore, developing therapeutic approaches for HSPs by targeting glial cells will utilize both stem cell-based models *in vitro* and animal models *in vivo*. Understanding the role of glial cells and the dynamic interactions between glial and neuronal cells is crucial for enhancing our comprehension of HSP and formulating effective therapeutic interventions.
